# A pilot study of depression among older people in Rawalpindi, Pakistan

**DOI:** 10.1186/1756-0500-7-409

**Published:** 2014-06-28

**Authors:** Farah Qadir, Sabahat Haqqani, Amna Khalid, Zille Huma, Girmay Medhin

**Affiliations:** 1 Department of Behavioural Sciences, Fatima Jinnah Women University, The Mall, Rawalpindi, Pakistan; 2 School of Health in Social Sciences, The University of Edinburgh, Edinburgh, UK; 3 Behavioural Sciences, Fatima Jinnah Women University, The Mall, Rawalpindi, Pakistan; 4 Aklilu Lemma Institute of Pathobiology, Addis Ababa University, P.O. Box 1176, Addis Ababa, Ethiopia

**Keywords:** Elderly mental health, Social support, Living arrangement

## Abstract

**Background:**

Depression is common among elderly in developed countries and it is more pronounced in institutional settings. In Pakistan there is a lack of empirical data on depression among this segment of the population particularly with reference to their living arrangements.

The objectives of the present study are to report the magnitude of depression among elderly having two different residential arrangements and to examine the association of depression and its established demographic factors.

**Findings:**

Data were collected from 141 respondents. 108 were community residents (m = 57 and f = 51) and 33 were living in the care homes (m = 29 and f = 4).

Prevalence of depression as assessed by Geriatric Depression Scale (GDS) among community and Care Homes (CHs) participants was 31.5 percent and 60.6 percent, respectively.

On Centre of Epidemiological Studies Depression Scale (CES-D), 42.6 percent of the community and 69.7 percent of the CH respondents were deemed depressed. Before adjusting for any other potential risk factors the odds of being depressed was significantly increased if the study participants were living in CH, relatively older, female, not currently married, had low educational level, had lower Mini Mental State Examination (MMSE) scores, and reported lower perceived emotional and practical support. In a partially adjusted logistic regression model an increased risk of depression was not confounded by any of the above mentioned risk factors.

However, the risk associated was not significant when it was adjusted for social support.

**Conclusions:**

The findings of the current study are consistent with previous research and throws light on the dire need for interventions to address mental health needs of Pakistani elderly.

Implications for improving the mental health status of elderly are also presented.

## Background

Depression is a common mental health concern in the older population [1]. By year 2020 depression will be the single leading cause of Disability Adjusted Life Years in the developing countries [2]. The prevalence of depression is 22.9 percent among Pakistani elderly as assessed by GDS (15 items) which indicates that every fifth elderly person over the age of 65 is suffering from depression [3]. However there is little research on this population particularly with reference to the roles of established risk factors like increased age, low socioeconomic status [4], gender [5], low education level, marital status [6], cognitive decline and social support [5].

Social support is seen as one of the determinants of health in the general population [5]. Lack of instrumental support has been associated with depression among older people, especially among those with higher levels of functional disability [7]. Asian and Pakistani families are considered traditionally as patriarchal, collectivist joint families harbouring three or more generations providing stable resilient and enduring support system. Any change in the structure would be likely to influence the harmony and support available in the original familial setup [8].

However, recent societal changes have not only affected their revered status in the family but also their position as the decision makers [9]. Due to the massive increase in urbanization rates in Asia its traditional multigenerational family system is being replaced by nuclear families, Pakistan is no different [10]. This decline in extended family system is also evident in Pakistan [8]. Consequently the personal space, autonomy and privacy replaces the constructs of empathy, reciprocity, and belongingness which in turn reduces the availability of support at home [11].

Like in most developing countries there is very little formal support available for older adults in Pakistan. Specialized geriatric care is almost unavailable. Care homes (CH) in larger cities are generally private or volunteer based providing services to vulnerable elderly. There is no published information about the quality of care and wellbeing of the elderly residing in such facilities [9]. Most of the Western studies indicate that those who reside in formal care units like CHs are more likely to have depression as compared to elderly residing in private homes [12-16]. Research indicates changes in living arrangements of elderly affect the structure of available support for them which in turn may influence self-rated mental health, physical health, and perceived social support [17]. It has also been reported that the increased social support suppresses the association of physical health decline and depression in older adults [18]. Although evidence for health problems and health care issues for the elderly is mounting over time, the role of social factors is rarely accounted for in geriatric health care research [19].

There is minimal investigation on depression among elderly and its relation with home versus institutionalized care in Pakistan. Keeping in mind the changing socio-demographic trends and paucity of evidence on depression among older Pakistani adults, this study aims to estimate and compare the prevalence of depression in relation to perceptions of support and other risk/protective factors among elderly residing in the community versus those residing in CHs. It is hypothesized that (1) prevalence of depression will be higher among elderly residing in institutions as compared to those residing at home, (2) perception of social support will be high among elderly residing in community as compared to those who reside in institutions, (3) after adjusting individually for age, gender, marital status and education, institutional residence (CH compared to family home) will be associated with increased likelihood of being depressed among the elderly.

## Method

### Study area

The data for the present study were collected from Rawalpindi district of Pakistan. Pakistan is a developing South Asian country and it is the sixth most populous country in the world [20]. The elderly population (60+ years) in this country was 6.1 percent in 2009 and is estimated to increase to 14.9 percent by 2050 [21]. This increase in number of elderly people in Pakistan in the absence of adequate medical and social care facilities warrants need assessment for appropriate measures to be taken [9].

### Participants

Study participants were recruited from CH and from the community. A total of 108 participants (57 males and 51 females) were selected from a residential colony in Rawalpindi city. Three CHs were approached, two were privately owned and one was managed by the Government. All participants who were present at the time of data collection and were able to take part in the research were interviewed with the exception of three male participants from the Government managed CH (Table 1). They were not willing to participate because they felt it was a futile effort and they seemed belligerent. It was therefore not deemed appropriate for the young female research assistants to pursue the elderly after the first attempt.

**Table 1:** Description of CH participants

**Care Home**	**Total Registered elderly**	**Total Interviewed**	**M**	**F**	**Reasons for not being interviewed**
Government	24	10	9	1	9 were not available
					2 participants were physically incapacitated
					3 did not consent
Private	5	3	2	1	2 participant was physically incapacitated
Private	20	20	17	3	-

The inclusion criteria was 60 or above years of age, willingness to take part in the study and ability to speak and understand Urdu. Those individuals who had severe physical health issues were excluded from the study.

### Data collection instruments

The protocol consisted of a demographic information sheet. The protocol also included Geriatric Depression scale (GDS) [22], The Centre for Epidemiologic Studies Depression Scale (CES-D) [23], Social support List of Interaction (SSLI-12) [24], and Mini Mental State Examination (MMSE) [25]. These instruments are briefly described as follows:

#### Demographic questionnaire

A short demographic questionnaire was developed to determine the demographic characteristics of the sample. Information on age, gender, family structure, marital status, employment status and living arrangements was obtained.

#### Geriatric Depression Scale (GDS)

This scale is one of the most widely used scales for assessing depressed mood among elderly [26]. It is composed of 30 self-rating items with a yes/no response option. Score of 0–10 indicates no depression, 11–20 indicates mild depression and 21–30 indicates severe depression [27]. This scale can be administered to healthy, medically ill and mild to moderate cognitively impaired elderly [28]. It has demonstrated 92 percent sensitivity and 89 percent specificity. Its psychometric properties have been found to be consistently good in clinical as well as research settings [29]. GDS-30 [30] and its shorter version GDS-15 [31] have been previously used successfully with Pakistani elderly population. Present study used the 30 item version of the scale.

#### The Centre for Epidemiologic Studies Depression Scale (CES-D)

This is a 20 item self-report scale to measure the levels of depressive symptoms based on the past week. The respondents are asked to rate the frequency of their symptoms on a scale of 0 (less than a day), 1 (1–2 days), 2 (3–4 days) and 3 (5–7 days) [32]. A cut-off value of 16 and above is used to report depressive symptoms [23]. CES-D has also been used in the Pakistani population [33].

#### Social Support List of Interaction (SSLI-12)

It measures the extent of social support from the primary social network as perceived by the elderly. It is a shorter version of social support list of interaction (34 items) developed by Van Sonderen [34]. It consists of 12 items, with likert scale scoring from 1 (*seldom or never*) to 4 (*very often*). It has three subscales that are; everyday support (emotional support), support in problems (practical support) and esteem support (support related to self-esteem) [24]. For the present study the first two subscales were used.

Items 1, 2, 3 and 6 measure the everyday support, items 4, 7, 8 and 9 measure support in problem situations and items 5, 10, 11 and 12 measure esteem support. In the present study, it was used as a continuous measure. The higher scores on SSLI-12 and its subscales suggest high level of perceived social support from the primary social network.

#### Mini Mental State Examination (MMSE)

This scale is composed of 11 questions and tasks, and it is one of the most widely used tests to screen cognitive impairment and to assess its severity [35]. Maximum score is 30, a score from 0–9 is indicative of severe cognitive impairment, score of 10–19 indicating moderate cognitive impairment, 20–25 indicating mild and 26–30 suggests no impairment. It has been used in various studies and has shown excellent psychometric properties in both clinical and research settings [36].

### Data collection procedure

All study participants were interviewed by two graduate researchers. Average time for one interview was 32.5 minutes. The two data collectors were trained for one day on how to conduct the interview by the lead author (FQ) of this paper.

### Ethical consideration

The ethical approval for the research was obtained from Fatima Jinnah Women University, Rawalpindi. Official permission was also sought from concerned authorities at the CHs. Participants were informed about the objective of the study. A written consent was obtained from participants of CHs and verbal consent was sought from the community participants.

### Data management and analysis

Data was computerized, cleaned and analysed using Statistical Package for Social Sciences version 13.0. Categorical variables were described using frequencies and continuous variables through means and standard deviations. The two measures of depression were used to evaluate the magnitude of depression and to assess the effect of hypothesized factors on the odds of being depressed. In logistic regression model the main exposure of interest was residential arrangement of elderly in which living within the community was considered as the reference category and living in the CH was considered as the category of interest. In the first step the effect of each of the target risk factors (residential arrangement, age, sex, current marital status, death of spouse, educational status, emotional support, practical support and MMSE score) was evaluated without adjusting for any other risk factor. In the second step the effect of residential arrangement was investigated after adjusting for each of the hypothesized risk factors (one risk factor at a time) and only the effect of residential arrangement was reported. Odds ratio and corresponding 95 percent confidence interval was reported as the measure of the strength of association of the target risk factor and the likelihood of depression.

Findings are reported as being statistically significant provided the p-value is less than 5 percent or the confidence interval around odds ratio does not include 1.

## Findings

Background characteristics of 141 study participants are summarized in Table 2. Their age ranges from 60–95 years with the mean age of 67.5 (SD = 6.94) years. There was no statistically significant difference in the mean age between those who were residing in the community (M = 67.2, SD = 6.51) and in old peoples home (M = 68.30, SD = 8.26) (p-value = 0.4396). Of the community sample 77.8 percent owned a home whereas only 4 of the 33 participants from the CHs sample were residing in their own home before getting institutionalized. Of the total study participants 41.8 percent (59/141) were widower/widow/separated (32.4 percent among community sample and 72.7 percent among CH sample). 54 (62.8%) men versus 28 (50.9%) women were married. However, 40 percent (N = 22) female elderly were widows as compared to 24.4 percent (N = 21) male elderly participants. Overall 75 percent of the community sample was unemployed. All the CH participants were unemployed, however, 58.6 percent reported pension as some source of income, 29.8 percent had a monthly income of less than 15000 Rs, and 23.4 percent were entirely dependent on their children for all financial matters. From the community sample 67.6 percent were living in joint family system and from the CH sample

**Table 2:** Socio-demographic characteristics of study participants (N = 141)

**Characteristics**	**Number (%) of Community Sample [108(76.6%)]**	**Number (%) of CH Sample [33(23.4%)]**	**Total number (%)**
**Employment Status**			
Currently employed	27(25)	0(0.0)	27(19.1)
Currently not employed	81(75)	33(100)	114(80.9)
**Ways of Earning**			
Self	71(65.7)	20(60.6)	91(64.5)
Others	37(34.3)	13(39.4)	50(35.5)
**Total Number of Children**			
Less than 3	65(60.2)	23(69.7)	88(62.4)
Three or more	43(39.8)	10(30.3)	53(37.6)
**Number of married children**			
None	16(14.8)	12(36.4)	28(19.9)
1-2 married children	48(44.4)	8(24.2)	56(39.7)
3 or more married children	44(40.8)	13(39.4)	57(40.4)
**No visit from offspring/s**	6(5.6)	17(51.5)	23(16.3)
**Who is your caregiver?**			
Spouse & Children	92(85.2)	4(12.1)	96(68.0)
Others	16(14.8)	29(87.9)	45(32)
**Have at least one caregiver**	106(98.1)	16(48.5)	122(86.5)
**Have servants at residential**	34(31.5)	6(18.2)	40(28.4)

51 percent were previously living in joint family system. Only 10.6 percent (5.6 percent community sample and 27.3 percent institutionalized) did not have children, 39.7 percent of the total study participants had 1–2 married children. Majority of the institutionalized participants reported that their children did not visit them at all.

The median (range) of GDS score was 19 (range 7 to 26) for the depressed group and 6 (range 0 to 10) for the non-depressed group. Similarly, the median (range) of CESD scores for the depressed group was 25 (range 16 to 57) and 10 (range 1 to 15) for the non-depressed group.

MMSE median score was 27 (range 12 to 30) for study participants residing within the community and 23 (range 9 to 30) for study participants residing in CHs.

In this sample a large proportion of depressed elderly reported decreased social support.

Among depressed elderly as assessed by CESD, 78.3 percent reported low emotional support and 69.6 percent reported low practical support. However, on GDS 81.5 percent and 72.2 percent of those where depressed elderly reported low emotional and practical support respectively. (See Table 3).

**Table 3:** Association of depression (as measured on CESD and GDS) with social support in terms of practical and emotional social support

**Type of Social support**	**Frequency and Percent depressed as measured with CESD**	**Frequency and Percent depressed as measured with GDS**	**P**
**Emotional support**			
Low	54(78.3)	44 (81.5)	0.00*
High	15 (21.7)	10 (18.5)	
**Practical support**			0.00*
Low	48 (69.6)	39(72.2)	
High	21(30.4)	15 (27.8)	0.00*

Note: *p < 0.05. The value of p corresponds to significance of association of both GDS and CESD with social support subscales. CESD = Centre for Epidemiologic Studies Depression Scale; GDS = Geriatric depression Scale.

The overall prevalence of depression in the current study based on CESD scores is 48.9 percent (n = 69/141) and 38.3 percent (n = 54/141) as assessed by GDS. The crude effects and partially adjusted effects of pre-specified risk factors including residential arrangement on the likelihood of depression among the elderly is summarized in Table 4 as measured by GDS and in Table 5 as measured by CESD. Depression was more frequent if the study participants were from the CH, if they were females, and if they did not have formal education. Before adjusting for other potential risk factors an increase in the odds of being depressed was significantly associated with CHs, female sex, currently being married, having lower educational level, lower score on emotional support scale, lower score on practical support scale and a lower score on MMSE scale. In the logistic regression model in which the effect of residential arrangement is partially adjusted for the selected potential confounding variable (i.e. one factor at a time) (see last columns of Tables 4 and 5) elderly people who live in CHs are more likely to be depressed. This effect was not confounded by any of the pre-specified risk factors except emotional and practical support that they received.

**Table 4:** The effect of living in CH on prevalence of depression (GDS) among elderly

**Characteristics of elderly study participants**	**Total number (%)**	**Number (%) depressed**	**Crude OR (95%CI)**	Partially adjusted OR (95%)*
Location				
Community	108(76.6)	34(31.5)	Ref	Ref
CH	33(23.4)	20(60.6)	3.35(1.49, 7.51)	3.35(1.49, 7.51)
*Age in years				
60-69	91(65.0)	36(39.6)	Ref	3.50(1.51, 8.12)
70+	49(35.0)	17(34.7)	0.81(0.39, 1.67)	
Sex				
Male	86(61.0)	26(30.2)	Ref	6.70(2.57, 17.43)
Female	55(39.0)	28(50.9)	2.39(1.19, 4.82)	
Current Marital status				
Married	82(58.2)	25(30.5)	Ref	2.77(1.18, 6.51)
Others	59(41.8)	29(49.2)	2.20(1.10, 4.41)	
Death of spouse				
Alive	89(63.1)	29(32.6)	Ref	2.96(1.28, 6.88)
Deceased	52(36.9)	25(48.1)	1.92(0.95, 3.86)	
Educational level				
At most can read and write	20(14.2)	12(60.0)	Ref	3.00(1.30, 6.93)
Grades 1-10	64(45.4)	29(45.3)	0.55(0.20, 1.53)	
Above grade 10	57(40.4)	13(22.8)	0.20(0.07, 0.58)	
Characteristics of elderly study participants	Number Interview	Mean (SD)	Crude OR (95%CI)	Partially adjusted OR (95%)*
Emotional support score	123	14.2(2.3)	0.74(0.62, 0.88)	2.11(0.76, 5.86)
Practical support score	122	6.9(1.7)	0.78(0.63, 0.98)	2.46(0.89, 6.76)
MMSE score	140	25.0(4.5)	0.89(0.82, 0.96)	2.44(1.03, 5.79)

*OR cross bonds to the effect of residence (CH versus community) after adjusting only for the corresponding background characteristic; SD = Standard deviation

Information of one respondent from CH is missing “age in years”.

**Table 5:** The effect of living in CH on prevalence of depression (CESD) among elderly

**Characteristics of elderly study participants**	**Total number (%)**	**Number (%) depressed**	**Crude OR (95%CI)**	Partially adjusted OR (95%)*
Location				
Community	108(76.6)	46(42.6)	Ref	Ref
CH	33(23.4)	23(69.7)	3.10(1.35, 7.14)	3.35(1.49, 7.51)
*Age in years				
60-69	91(65.0)	41(45.1)	Ref	2.83(1.21, 6.61)
70+	49(35.0)	27(55.1)	1.50(0.74, 3.01)	
Sex				
Male	86(61.0)	36(41.9)	Ref	5.04(2.01, 12.63)
Female	55(39.0)	33(60.0)	2.08(1.05, 4.15)	
Current Marital status				
Married	82(58.2)	31(37.8)	Ref	2.25(0.93, 5.46)
Others	59(41.8)	38(64.4)	2.98(1.49, 5.97)	
Death of spouse				
Alive	89(63.1)	38(42.7)	Ref	2.68(1.13, 6.39)
Deceased	52(36.9)	31(59.6)	1.98(0.99, 3.97)	
Educational level				
At most can read and write	20(14.2)	15(75.0)	Ref	2.76(1.16, 6.58)
Grades 1-10	64(45.4)	36(56.2)	0.43(0.14, 1.32)	
Above grade 10	57(40.4)	18(31.6)	0.15(0.05, 0.49)	
Characteristics of elderly study participants	Number interviewed	Mean (SD)	Crude OR (95%CI)	Partially adjusted OR (95%)*
Emotional support score	123	14.2(2.3)	0.69(0.57, 0.84)	1.80(0.63, 5.13)
Practical support score	122	6.9(1.7)	0.70(0.54, 0.90)	1.62(0.58, 4.58)
MMSE score	140	25.0(4.5)	0.87(0.80, 0.95)	2.12(0.87, 5.17)

*OR cross bonds to the effect of residence arrangement (CH versus community) after adjusting only for the corresponding background characteristic; SD = Standard deviation.

Information of one respondent from CH is missing “age in years”.

## Discussion

The present study compared frequency of depression and its correlates among Pakistani elderly aged 60 years and above residing either in the community or in CH. To the best of our knowledge this comparison has not been examined in this population before.

In our study the prevalence of depression in the community elderly was 31.5 percent and 42.6 percent based on GDS and CES-D scores respectively. Our community prevalence rates are consistent with one of the previous cross- sectional studies carried out in Karachi in which the prevalence using the shorter version of GDS was (40 percent) [37]. Within the region, community prevalence for depression in an Indian study was reported to range from 11.6 percent to 31.1 percent [38]. However, our rates are considerably higher than 22.9 percent reported by Ganatra and colleagues [3]. In the present study, GDS and CESD scores for CH participants (60.6 percent and 69.7 percent respectively) are disproportionately higher compared to studies carried out in other parts of the world [5,39]. A review based on 122 studies in the West reported the prevalence of major depression between the range of 0.9 percent to 9.4 percent in private households and from 14 percent to 42 percent in institutional settings [5]. Whereas a UK based study on residents in CHs found 40 percent prevalence [39]. The prevalence rates have varied in different studies and the disparity has been ascribed to the clinical criteria used to measure depression, the cultural settings and the population diversity [40].

There is robust evidence indicating higher rates of depression in CHs compared to the community [13-16]. Au contraire, studies have also indicated that residents of CHs are less depressed compared to those residing in their own homes [41]. Among the consistent correlates is CH resident’s perception of support, companionship, and safe environment [41].

Although the overall results of the study indicate a high prevalence of depression among elderly in both groups, people living in CHs are at an increased risk of depression compared to their counterparts. One plausible explanation for the high prevalence rates could be the challenge faced by Pakistan in dealing with the aging population which is estimated to increase to 14.8 percent by 2050 from 6.5 percent in 2013 [42]. The country’s health system has inadequate resources [9] and lacks the preparedness to meet the needs of the elderly who are pivotal in the social and familial structure of the society. In the context of Pakistan it is imperative to understand the perspective of the elderly in the familial and social context. The family has been consistently reported to be a source of strength and security for the elderly in providing them with social, financial and emotional support [43,44] which is associated with their well-being. Therefore it is not surprising that study participants who were residents of CHs reported higher rates of depression perhaps attributable to their feeling of isolation from their families [45,46].

The other reason for higher prevalence in the current study could be that most of our study participants were in the age range of 60–75 years (younger elderly) and depression is reported to be more common among younger elderly [47]. Further research is recommended to better understand these associations.

In our logistic regression analysis which adjusted only for social support; residential arrangement was not a significant predictor for the probability of depression among elderly.

Moreover, in the unadjusted model increased social support did have a protective effect against depression. The differential risk between residential arrangements was not significant after adjusting individually for emotional and practical support. Risk of depression was also independently associated with reduced support and lower MMSE score before controlling for the effect of respondent’s living arrangement. These findings are consistent with an established association between various sources of support and depression among elderly [5]. Several studies revealed that family support was the most effective factor influencing the sense of depression experienced by residents of CHs [47-50].

Although our study due to its limited sample does not allow a conclusive estimate, it does however, contribute to the repertoire of research identifying an increasing urgent need to address the mounting problems related to the well-being of elderly in Pakistan in order to inform policy makers to address the needs of this neglected population. However, we need to bear in mind that neither GDS nor CESD generates a clinical diagnosis; therefore it is imperative to carry out further research to establish a more explicit diagnosis.

Some of the existing evidence shows that depression decreases with age among elderly CH residents [47] while other studies report that there is no significant association between age and depression in CH residents [51]. In the present study there was no significant association between age and likelihood of depression and the positive effect of being resident of CH on the likelihood of depression was not confounded by age, sex, current marital status, death of spouse and educational status. Currently married elderly have reduced risk of being depressed which is also in agreement with previous research [6].

Our study does not have an equal proportion of female representation in the CH sample therefore, although the findings show that male CH respondents are more likely to be depressed we must interpret the results with caution. Further research must be carried out to explore this association which is in contrast with the previous findings [5]. It is perhaps not surprising that there were very few females in CHs. According to the cultural milieu of Pakistan the sin qua non for elderly is to be looked after by their families in old age. Therefore even under dire circumstances it is less likely for the families to send their female elderly to CHs, and just as improbable for them to leave unpleasant familial circumstances and volunteer for a CH living arrangement. This is in accordance with Pakistan being a patriarchal society where families are less likely to allow women to leave and seek refuge outside of home. It might also be plausible that relatives are more likely to provide support to female elderly as compared to males. This is in contrast to West where more females than males are likely to go to care homes because they marry older men who die earlier [52]. In the current study there was no significant association between depression and death of spouse before adjusting for respondent’s residence which is in contrast to the literature suggesting that spousal bereavement is a significant risk factor for late life depression [53]. The possible reason for this could be that the elderly spouse might not be able to provide adequate level of support. Furthermore, in the current study we do not have information about the marital relationships of the respondents. The literature supports that people who are not happy with their relationships are more likely to be depressed as compared to those without partners [54]. The fact that CH respondents with living spouse had a higher chance of depression might be explained in terms of them missing their spouses and feeling that they were better off with their spouses as compared to living alone in CH. In future studies this feeling of abandonment should be explored further.

Before adjusting for residential arrangement having higher score on MMSE was protective against depression and the MMSE score did not confound the positive effect of living in CH on the likelihood of depression as measured by GDS and CESD. This was consistent with literature which indicates higher cognitive impairment as a significant predictor of depression among elderly [55].

In conclusion our study takes a small step towards establishing and recognizing the prominent role of familial support in later life. The demographic changes and other contributing factors that are challenging the revered position of the elderly in Pakistan resulting in their displacement from home to CHs and its association with their mental health needs to be addressed in research. It is essential to develop indigenously appropriate interventions.

### Limitations

One of the limitations of the current study is the cross-sectional study design which does not establish the direction of causality. In the current study it would be difficult to make a firm conclusion about whether depressed elderly are more likely to go to CH or being institutionalized leads to depression. The snow ball sampling technique limits the population representation. This technique was used to facilitate access to the elderly who are likely to be cared for at homes in Pakistan and are difficult to access without a local reference. The socio-political situation in Pakistan does not allow unfamiliar person entering the house without any reference and the probability of a male answering the door is higher. The small sample size of the study has a potential effect on generalizability of the results. The use of self-report measures and fewer female participants in CHs contribute to potential bias. In the current study a full logistic regression model adjusting for all risk factors simultaneously on the effect of residential setting was not feasible due to the small sample size and the observed effect might have been an overestimate of the true effect.

### Implications

The results of the present study suggest that for effective elderly care arrangements socio-demographic characteristics must be taken into account as these play a significant role in elderly mental health. They highlight the importance of providing consistent social support to facilitate transition from community to CH. Thus this study despite its limitations provides important preliminary evidence for designing health care facilities intended for elderly in Pakistan.

## Abbreviations

CH/ CHs: Care Home/s; GDS: Geriatric Depression scale [22]; CES-D: The Centre for Epidemiologic Studies Depression Scale [23]; SSLI-12: Social support List of Interaction [24]; MMSE: Mini Mental State Examination [25].

## Competing interests

There are no competing interests.

## Authors' contributions

FQ: Conceived the research idea, supervised data collection, drafted the MS, and contributed to the interpretation of the findings. GM: Contributed to the analysis, write-up and interpretation of the findings. AK: Contributed to the write-up and interpretation of the findings. ZH: Contributed to the analysis of data and write-up of the results. SH: Contributed to the write-up of the MS and interpretation of the findings. All authors read and approved the final manuscript.

## References

[B1] U.S. Department of Health and Human Services Older adults and mental health. In: mental health: a report of the surgeon general 1999 http://www.surgeongeneral.gov/library/mentalhealth/chapter5/sec1.html

[B2] Murray CJ Lopez AD Alternative projections of mortality and disability by cause 1990–2020: global burden of disease study Lancet 1997 349 1498 1504 9167458 10.1016/S0140-6736(96)07492-2

[B3] Ganatra HA Zafar SN Qidwai W Rozi S Prevalence and predictors of depression among an elderly population of Pakistan Aging Ment Health 2008 12 3 349 356 18728948 10.1080/13607860802121068

[B4] Blazer D Burchett B Service C George LK The association of age and depression among the elderly: an epidemiological exploration J Gerontol 1991 46 6 M210 M215 1834726 10.1093/geronj/46.6.m210

[B5] Djernes JK Prevalence and predictors of depression in populations of elderly: a review Acta Psychiatr Scand 2006 113 372 387 16603029 10.1111/j.1600-0447.2006.00770.x

[B6] Luppa M Sikorski C Luck T Weyerer S Villringer A Konig HH Riedel-Heller SG Prevalence and risk factors of depressive symptoms in latest life–results of the Leipzig Longitudinal Study of the Aged (LEILA 75+) Int J Geriatr Psychiatry 2012 27 3 286 295 21538535 10.1002/gps.2718

[B7] Prince MJ Harwood RH Blizard RA Thomas A Mann AH Social support deficits, loneliness and life events as risk factors for depression in old age. The Gospel Oak Project VI Psychol Med 1997 27 2 323 332 9089825 10.1017/s0033291796004485

[B8] Itrat A Taqui AM Qazi F Qidwai W Family systems: perceptions of elderly patients and their attendants presenting at a university hospital in Karachi, Pakistan J Pak Med Assoc 2007 57 2 106 110 17370799

[B9] Sabzwari S Azhar G Ageing in Pakistan – a new challenge Ageing International 2010 doi: 10.1007/s12126-010-9082-z

[B10] Bongaarts J Household size and composition in the developing world, series: working papers, numb 144 http://popcouncil.org/pdfs/wp/144.pdf

[B11] Alam M Ageing in India: socio-economic and health dimensions 2006 New Delhi: Academic Foundation

[B12] Snowdon J Lane F The prevalence and outcome of depression and dementia in Botany’s elderly population Int J Geriatr Psychiatry 2001 16 3 293 299 11288164 10.1002/gps.339

[B13] Rovner B Prevalence of mental illness in a community nursing home Am J Psychiatr 1986 143 1446 1449 3777239 10.1176/ajp.143.11.1446

[B14] Gorey KM Cryns AG Group work as interventive modality with the older depressed client: a meta-analytic review J Gerontol Soc Work 1991 16 1/2 137 157

[B15] Grayson P Lubin B Whitlock R Comparison of depression in the community-dwelling and assisted-living elderly J Clin Psychol 1995 51 1 18 21 7782469 10.1002/1097-4679(199501)51:1<18::aid-jclp2270510104>3.0.co;2-w

[B16] Ron P Depression, hopelessness, and suicidal ideation among the elderly: a comparison between men and women living in nursing homes and in the community J Gerontol Soc Work 2004 43 2/3 97 116

[B17] Zafar SN Ganatra HA Tehseen S Qidwai W Health and needs assessment of geriatric patients: results of a survey at a teaching hospital in Karachi, Pakistan J Pak Med Assoc 2006 56 470 474 17144398

[B18] Suttajit S Punpuing S Jirapramukpitak T Tangchonlatip K Darawuttimaprakorn N Stewart R Dewey ME Prince M Abas MA Impairment, disability, social support and depression among older parents in rural Thailand Psychol Med 2010 40 10 1711 1721 20056022 10.1017/S003329170999208X PMC2928999

[B19] Patel V Prince M Ageing and mental health in a developing country: who cares? Qualitative studies from Goa, India Psychol Med 2001 31 29 38 11200957 10.1017/s0033291799003098

[B20] Population Reference Bureau The 2006 world health data sheet http://www.prb.org/pdf06/06WorldDataSheet.pdf

[B21] United Nation World population prospects the 2008 revision: highlights 2009 New York: Department of Economic and Social Affairs, Population Division

[B22] Yesavage JA Brink TL Rose TL Lum O Huang VS Adey M Leirer VO Development and validation of a geriatric depression screening scale: a preliminary report J Psychiatr Res 1983 17 37 39 7183759 10.1016/0022-3956(82)90033-4

[B23] Radloff LS The CES-D scale: a self-report depression scale for research in the general population Appl Psychol Meas 1977 1 3 385 401

[B24] Kempen GIJM Van Eijk LM The psychometric properties of the SSL12-I, a short scale for measuring social support in the elderly Soc Indic Res 1995 35 3 303 312

[B25] Folstein MF Folstein SE McHugh PR Mini-mental state”. A practical method for grading the cognitive state of patients for the clinician J Psychiatr Res 1975 12 189 198 1202204 10.1016/0022-3956(75)90026-6

[B26] Montorio I Izal M The geriatric depression scale: a review of its development and utility Int Psychogeriatr 1996 8 103 112 8805091 10.1017/s1041610296002505

[B27] Debruyne H van Buggenhout M Le Bastard N Aries M Audenaert K De Deyn PP Engelborghs S Is the geriatric depression scale a reliable screening tool for depressive symptoms in elderly patients with cognitive impairment? Int J Geriatr Psychiatry 2009 24 6 556 562 19132643 10.1002/gps.2154

[B28] Kurlowicz L Greenberg SA The Geriatric Depression Scale (GDS) AJN 2007 107 10 67 68

[B29] Mitchell AJ Bird V Rizzo M Meader N Which version of the geriatric depression scale is most useful in medical settings and nursing homes? Diagnostic validity meta-analysis Am J Geriatr Psychiatry 2010 18 1066 1077 21155144 10.1097/jgp.0b013e3181f60f81

[B30] Mokhber N Gharavi MM Comparison of nortriptyline and bupropion in major depressive disorder among elderly patients J Pak Psychiatr Soc 2007 4 2 83

[B31] Sheikh JI Yesavage JA Brink TL Geriatric Depression Scale (GDS) Recent evidence and development of a shorter version Clinical gerontology: a guide to assessment and intervention 1986 New York: The Haworth Press 165 173

[B32] Cheng ST Chan AC The center for epidemiologic studies depression scale in older Chinese: thresholds for long and short forms Int J Geriatr Psychiatry 2005 20 5 465 470 15852439 10.1002/gps.1314

[B33] Kazi A Fatmi Z Hatcher J Kadir MM Niaz U Wasserman GA: social environment and depression among pregnant women in urban areas of Pakistan: importance of social relations Soc Sci Med 2006 63 1466 1476 16797813 10.1016/j.socscimed.2006.05.019

[B34] Van Sonderen FLP The measurement of social support with the SSQ-I and the SSQ-D. Duct manual 1993 Groningen: Noordelijk Centrum voor Gezondheidsvraagstukken

[B35] Pangman VC Sloan J Guse L An examination of psychometric properties of the mini-mental state examination and the standardized mini-mental state examination: implications for the clinical practice Appl Nurs Res 2000 13 209 213 11078787 10.1053/apnr.2000.9231

[B36] Kaasalainen S Middleton J Knezacek S Hartley T Stewart N Ife C Robinson L Pain and cognitive status in the institutionalized elderly: perceptions and interventions J Gerontol Nurs 1998 24 8 24 31 9782869 10.3928/0098-9134-19980801-07

[B37] Bhamani MA Karim MS Khan MM Depression in the elderly in Karachi, Pakistan: a cross sectional study BMC Psychiatry 2013 13 181 23819509 10.1186/1471-244X-13-181 PMC3704964

[B38] Barua A Ghosh MK Prevalence of depressive disorders in the elderly Annuals of Saudi Medicine 2011 31 6 620 624 10.4103/0256-4947.87100 PMC3221135 22048509

[B39] Mann AH Schneider J Mozley CG Levin E Blizard R Netten A Kharicha K Egelstaff R Abbey A Todd C Depression and the response of residential homes to physical health needs Int J Geriatr Psychiatry 2000 15 1105 1112 11180466 10.1002/1099-1166(200012)15:12<1105::aid-gps252>3.0.co;2-w

[B40] Evans M Mottram P Diagnosis of depression in elderly patients Adv Psychiatr Treat 2000 6 1 49 56

[B41] Chung S Residential status and depression among Korean elderly people: a comparison between residents of nursing home and those based in the community Health Soc Care Community 2008 16 4 370 377 18613913 10.1111/j.1365-2524.2007.00747.x

[B42] United Nations: World Population Prospects The 2012 Revision, Key Findings and Advance Tables 2013 New York: Department of Economic and Social Affairs, Population Division

[B43] Kramer EJ Kwong K Lee E Chung H Cultural factors influencing the mental health of Asian Americans West J Med 2002 176 4 227 12208826 PMC1071736

[B44] Bongaarts J Household size and composition in the developing world in the 1990s Popul Stud 2001 55 263 279

[B45] Cano A Scaturo DJ Sprafkin RP Lantinga LJ Fiese BH Brand F Family support, self rated health and psychological distress: primary care companion J Clin Psychiatry 2003 5 111 117 10.4088/pcc.v05n0302 PMC406377 15154021

[B46] Marino P Sirey JA Raue PJ Alexopolous GS Impact of social support and self efficacy on functioning in depressed older adults with chronic obstructive pulmonary disease Int J COPD 2008 3 4 713 718 10.2147/copd.s2840 PMC2650598 19281085

[B47] Burvill PW Hall WD Stampfer HG Emmerson JP The prognosis of depression in old age Br J Psychiatry 1991 158 64 71 2015452 10.1192/bjp.158.1.64

[B48] Green BH Copelan JR Dewey ME Sharma V Saunders PA Davidso IA Risk factors for depression in elderly people: a prospective study Acta Psychiatr Scand 1992 86 213 217 1414415 10.1111/j.1600-0447.1992.tb03254.x

[B49] Peters A Liefbroer AC Beyond marital status: partner history and well-being in old age J Marriage Fam 1997 59 687 699

[B50] Chung S A path analysis on depression among the elderly of institutionalized settings J Korean Gerontol Soc 2005 25 3 75 90

[B51] Jongenelis K Pot AM Eisses AM Beekman AT Kluiter H Ribbe MW Depression among older nursing home patients. A review Tijdschr Gerontol Geriatr 2003 34 52 59 12741088

[B52] Borland S Women 40% more likely than men to go into care homes (because they marry older men who die earlier). Daily mail 2012 Retrieved from http://www.dailymail.co.uk/news/article-2131296/Women-40-likely-men-care-homes.html

[B53] Byrne G Raphael B A longitudinal study of bereavement phenomena in recently widowed elderly men Psychol Med 1994 24 411 21 8084936 10.1017/s0033291700027380

[B54] Ross CE Reconceptualising marital status as a continuum of social status J Marriage Fam 1995 57 1 129 140

[B55] Vinkers DJ Gussekloo J Stek ML Westendorp RGJ Van der Mast RC Temporal relation between depression and cognitive impairment in old age: prospective population based study Br Med J 2004 329 7471 881 883 15345599 10.1136/bmj.38216.604664.DE PMC523108

